# Correction: A fast and robust interpolation filter for airborne lidar point clouds

**DOI:** 10.1371/journal.pone.0233128

**Published:** 2020-05-07

**Authors:** Chuanfa Chen, Yanyan Li, Na Zhao, Jinyun Guo, Guolin Liu, the PLOS ONE Editors

## Notice of Republication

The authors would like to clarify that in the published article [1], the proposed classification method is an improvement of their previous method of multi-resolution hierarchical classification (MHC) which used computationally costly analytical thin plate spline (TPS) for interpolating initial ground points [2]. The improved classification method [1] replaced the interpolated values with the original values, and used discrete cosine transform (DCT)-based finite difference TPS, as previously developed by Damien Garcia in [3], to perform interpolations only where no lidar points were located. The *PLOS ONE* article does not present a new interpolation method; basic information about the existing interpolation methods of analytical TPS and (weighted) finite difference TPS was included in the article for completeness.

The authors acknowledge that the discussion of previous work by Dr. Garcia [3] referred only to computational complexity, and did not clearly state that the reported new classification method incorporated the use of interpolation methods originally published by Dr. Garcia.

The authors hereby clarify that in the Fast and robust TPS for gridded surface interpolation subection of the Principle of the proposed method for lidar point classification section, the DCT method originally published by Dr. Garcia was used to solve the linear equation of finite difference TPS (i.e. Eq 7 and Eq 10). Note that to solve Eq 7 and Eq 10, the authors used the MATLAB codes DCTN, IDCTN and SMOOTHN [3], [4] available from the MATLAB file exchange via the following links:

https://cn.mathworks.com/matlabcentral/fileexchange/25634-robust-spline-smoothing-for-1-d-to-n-d-data
https://cn.mathworks.com/matlabcentral/fileexchange/26040-dct-and-dst--+-inverse--in-arbitrary-dimension

The authors apologize for the omission of reference [4], as required in the re-use of the SMOOTHN MATLAB code.

The copyright for the original MATLAB codes SMOOTHN, DCTN, and IDCTN belongs to Dr. Garcia and the codes are distributed under the terms of a BSD license, which requires that the license remains attached to the code and included in any derivative work. The original version of this article provided the MATLAB codes in S1 File, but did not include the license for these MATLAB codes; as a result of the breach of the license terms, the article has been republished with **the MATLAB codes SMOOTHN, DCTN, and IDCTN** removed from the Supporting Information, and the authors instead refer readers to the links above.

The above-mentioned issues are corrected in the republished version of the article. The original version of the article text is included here as a Supporting Information file. The following is a list of changes incorporated in the republished article:

- A new sentence was added after the third sentence of the first paragraph of the Principle of the proposed method for lidar point classification. The new sentence is: The proposed method incorporates the existing interpolation methods analytical TPS [35] and weighted finite difference TPS [25]. The following sections provide background information about these existing interpolation methods.
- References 25 and 36 were added to the sentences immediately following Eq 6 and Eq 9 in the Fast and robust TPS for gridded surface interpolation subsection of Principle of the proposed method for lidar point classification.
- A new sentence was added to the end of the final paragraph of the Fast and robust TPS for gridded surface interpolation subsection of Principle of the proposed method for lidar point classification. The new sentence is: Note that to solve Eq 7 and 10, we use the MATLAB codes DCTN, IDCTN and SMOOTHN [25, 36] available from the MATLAB file exchange via the following links:
  1. https://cn.mathworks.com/matlabcentral/fileexchange/25634-robust-spline-smoothing-for-1-d-to-n-d-data
  2. https://cn.mathworks.com/matlabcentral/fileexchange/26040-dct-and-dst--+-inverse--in-arbitrary-dimension.
- A URL was added to the first sentence of the Experiments and Results. The correct sentence is: Fifteen benchmark reference samples from seven sites, provided by ISPRS Commission III/WG3 [7] and downloaded from the website (https://www.itc.nl/isprs/wgIII-3/filtertest/downloadsites/), were used to assess the performances of the proposed algorithm and MHC.
- A new reference was added to the References. The new reference is: 36. Garcia D. A fast all-in-one method for automated post-processing of PIV data. Experiments in Fluids. 2011;50(5):1247–59. doi: 10.1007/s00348-010-0985-y.

## Supporting information

S1 File Originally published, uncorrected article. (PDF)

S2 File Republished, corrected article. (PDF)
