# Supplementary material for: Correction: A fast and robust interpolation filter for airborne lidar point clouds
Source: PLoS One. 2020 May 7;15(5):e0233128. doi: 10.1371/journal.pone.0233128 (PMC7205228; doi:10.1371/journal.pone.0233128)
Supplement: S2 File — (PDF) [file pone.0233128.s002.pdf]

PLOS ONE

RESEARCH ARTICLE

# A fast and robust interpolation filter for airborne lidar point clouds

**Chuanfa Chen[1,2]*, Yanyan Li[3], Na Zhao[4], Jinyun Guo[1,2], Guolin Liu[2]**

**1** State Key Laboratory of Mining Disaster Prevention and Control Co-founded by Shandong Province and the Ministry of Science and Technology, Shandong University of Science and Technology, Qingdao, China, **2** Shandong Provincial Key Laboratory of Geomatics and Digital Technology of Shandong Province, Shandong University of Science and Technology, Qingdao, China, **3** Shool of Geodesy and Geomatics, Wuhan University, Wuhan, China, **4** State Key Laboratory of Resources and Environment Information System, Institute of Geographical Sciences and Natural Resources Research, Beijing, China

* chencf@lreis.ac.cn

Check for updates

OPEN ACCESS

**Citation:** Chen C, Li Y, Zhao N, Guo J, Liu G (2017) A fast and robust interpolation filter for airborne lidar point clouds. PLoS ONE 12(5): e0176954. https://doi.org/10.1371/journal.pone.0176954

**Editor:** Juan A. Añel, Universidade de Vigo, SPAIN

**Received:** November 7, 2016

**Accepted:** April 19, 2017

**Published:** May 3, 2017

**Copyright:** © 2017 Chen et al. This is an open access article distributed under the terms of the Creative Commons Attribution License, which permits unrestricted use, distribution, and reproduction in any medium, provided the original author and source are credited.

**Data Availability Statement:** All relevant data are within the paper and its Supporting Information files.

**Funding:** This work is supported by National Natural Science Foundation of China (Grant No. 41371367), SDUST Research Fund, Joint Innovative Center for Safe and Effective Mining Technology and Equipment of Coal Resources, Shandong Province, and Special Project Fund of Taishan Scholars of Shandong Province.

**Competing interests:** The authors have declared that no competing interests exist.

## Abstract

A fast and robust interpolation filter based on finite difference TPS has been proposed in this paper. The proposed method employs discrete cosine transform to efficiently solve the linear system of TPS equations in case of gridded data, and by a pre-defined weight function with respect to simulation residuals to reduce the effect of outliers and misclassified non-ground points on the accuracy of reference ground surface construction. Fifteen groups of benchmark datasets, provided by the International Society for Photogrammetry and Remote Sensing (ISPRS) commission, were employed to compare the performance of the proposed method with that of the multi-resolution hierarchical classification method (MHC). Results indicate that with respect to kappa coefficient and total error, the proposed method is averagely more accurate than MHC. Specifically, the proposed method is 1.03 and 1.32 times as accurate as MHC in terms of kappa coefficient and total errors. More importantly, the proposed method is averagely more than 8 times faster than MHC. In comparison with some recently developed methods, the proposed algorithm also achieves a good performance.

## Introduction

With an efficient collection of high-resolution 3D information of the Earth's surface, airborne light detection and ranging (lidar) data have been widely used in many applications, such as construction of digital elevation models (DEMs) [1], forest inventory [2, 3] and animal distribution simulation [4]. Since raw lidar data contains a large volume of points acquired from different objects [5, 6], it is necessary to differentiate ground and non-ground points.

Many filtering algorithms have been proposed to extract ground points from raw point clouds. Generally, these methods can be categorized into three main groups [7–9]: slope-based, morphological-based and interpolation-based filters. Slope-based methods are based on the assumption that two nearby points should have a small height difference. Thus, if the slope of two nearby points is larger than a predefined threshold, the higher elevation point is classified as the non-ground point [10]. However, due to constant slope threshold, slope-based

methods restrict their uses to smooth terrain. Morphological-based filters employ a series of operations, such as opening and closing, on lidar measurements to approximate terrain surface. The success of this method mainly depends on the selection of a proper filter window size. Ideally, the window size should be small enough to preserve subtle terrain features and large enough to remove non-ground objects [11–15]. However, it is difficult to determine the optimal size in practice. For interpolation-based filters, a critical step is to construct reference surfaces using interpolation methods. Thin plate spline (TPS) with high interpolation accuracy and numerical stability has been commonly adopted [16–19]. It is generally performed with an analytical version. However, the analytical TPS has a highly computational cost due to its local interpolation of the huge volume of data points for surface construction [18–23]. Alternatively, the reference surface can be globally and efficiently produced by finite difference TPS in case of gridded data [24]. For example, the computational complexity of solving a linear system with discrete cosine transform (DCT) is only $O(n \log(n))$, whereas that of analytical TPS is $O(n^3)$, where $n$ is the order of the system [25].

Motivated by this idea, a fast and robust interpolation filter based on finite difference TPS is developed in this paper. Compared with the present TPS-based filters [16–19], the advantages of the proposed method are as follows: (i) it is computationally efficient, as DCT is employed to solve the linear system of TPS equations; and (ii) it is robust, since a pre-defined weight function with respect to fitting residuals is introduced to resist the effect of outliers and non-ground points on the construction of reference ground surfaces.

## Principle of the proposed method for lidar point classification

The proposed filter can be considered as an updated version of multi-resolution hierarchical classification (MHC) algorithm [18], which is grouped into interpolation-based filters. Like MHC, the new method uses a hierarchy with three levels, where the resolution of reference surfaces steadily increases from the low to the high level. Unlike MHC, the proposed method achieves surface interpolation with a robust finite difference TPS. The proposed method incorporates the existing interpolation methods analytical TPS [35] and weighted finite difference TPS [25]. The following sections provide background information about these existing interpolation methods.

### Analytical TPS for surface interpolation

Let $(x_i, y_i, z_i)$, $i = 1,\ldots, n$ represent the coordinates of airborne lidar points. Suppose that the data set can be modeled as $z_i = f(x_i, y_i) + e_i$, where $e_i$ is an independent and normally distributed noise with zero mean and unknown variance; $f(x, y)$ is a smoothing function used to describe the surface.

TPS interpolation is achieved by minimizing a criterion function that balances the tradeoff between the fidelity to the data and the smoothness of the interpolated surface [26]. Specifically, the objective function of TPS is expressed as

$$\min_f (\|z_i - f(x_i, y_i)\|^2 + \lambda T(f)) \quad (1)$$

where $\lambda$ is a smoothing parameter determined by the generalized cross validation; $T(f)$ represents the penalty term of the smoothness defined as $T(f) = \int_{R^2} (f_{xx}^2 + 2f_{xy}^2 + f_{yy}^2)dxdy$, where $f_{xx}$ and $f_{yy}$ are the second order partial derivatives of $f(x, y)$ with respect to $x$ and $y$, respectively, and $f_{xy}$ is the cross partial derivative.

For the analytical TPS, the function is expressed as,

$$f(x_0, y_0) = \sum_{i=1}^{t} q(r_{0i})\alpha_i + \sum_{j=0}^{s} p_j(x, y)\beta_j \tag{2}$$

where $q(\cdot)$ and $\alpha$ represent the kernel function and the corresponding weight; $p(\cdot)$ and $\beta$ represent the polynomial and its coefficient; $r_{0i}$ is the Euclidean distance between the interpolated point $(x_0, y_0)$ and the $i$th sample point; $t$ and $s$ represent the number of sample points used for computation and the order of the polynomial; $q(r) = r^2\log(r)$.

To estimate the surface, the parameters $\alpha$ and $\beta$ must be pre-obtained by solving the following system:

$$\begin{bmatrix} \boldsymbol{Q} + \lambda \boldsymbol{I} & \boldsymbol{P} \\ \boldsymbol{P}^T & \boldsymbol{0} \end{bmatrix} \begin{bmatrix} \boldsymbol{\alpha} \\ \boldsymbol{\beta} \end{bmatrix} = \begin{bmatrix} \boldsymbol{z} \\ \boldsymbol{0} \end{bmatrix} \tag{3}$$

where $\boldsymbol{Q} = \begin{bmatrix} q_{11} & \cdots & q_{1t} \\ \vdots & \ddots & \vdots \\ q_{t1} & \cdots & q_{tt} \end{bmatrix}$; $q_{ij} = q(r_{ij})$; $\boldsymbol{P} = \begin{bmatrix} p_{10} & \cdots & p_{1s} \\ \vdots & \ddots & \vdots \\ p_{t0} & \cdots & p_{ts} \end{bmatrix}$; $p_{ij} = p_j(x_i, y_i)$.

Generally, the computing cost for solving (Eq 3) is about $t^3/3$ [27]. In practice, the analytical TPS is commonly performed within a local neighborhood. Supposing the number of neighbors for an interpolation is $k$, the cost of the local TPS for estimating a surface with $m\times n$ grids is about $(k^3mn)/3$. Thus, the analytical TPS with a local interpolation still needs a highly computational cost.

## Fast and robust TPS for gridded surface interpolation

In case of gridded surface, $T(\boldsymbol{f})$ can be formulated by a second-order finite difference operator [28]:

$$T(\boldsymbol{f}) = \|\boldsymbol{D}\boldsymbol{f}\|^2 \tag{4}$$

where $\boldsymbol{f} = [f_{11} \cdots f_{1m}; f_{21} \cdots f_{2m}; \cdots; f_{n1} \cdots f_{n\text{-}m}]^T$; $f_{ij}$ is the function value at the grid $(i, j)$; $n$ and $m$, respectively, denote the row and column of gridded data; $\boldsymbol{D} = [\boldsymbol{D}_1, 2\boldsymbol{D}_2, \boldsymbol{D}_3]^{\mathrm{T}}$; $\boldsymbol{D}_1$, $\boldsymbol{D}_2$ and $\boldsymbol{D}_3$ represent the second-order finite difference matrix of $f_{xx}$, $f_{xy}$ and $f_{yy}$, respectively.

Based on (Eq 4), the matrix form of (Eq 1) is expressed as:

$$\min_{f} (\boldsymbol{z} - \boldsymbol{f})^T(\boldsymbol{z} - \boldsymbol{f}) + \lambda \boldsymbol{f}^T\boldsymbol{D}^T\boldsymbol{D}\boldsymbol{f} \tag{5}$$

Minimizing (Eq 5) with respect to $\boldsymbol{f}$ and letting it be zero, we can obtain the equation: $\boldsymbol{f} - \boldsymbol{z} + \lambda\boldsymbol{D}^T\boldsymbol{D}\boldsymbol{f} = \boldsymbol{0}$. Namely, $(\boldsymbol{I} + \lambda\boldsymbol{D}^T\boldsymbol{D})\boldsymbol{f} = \boldsymbol{z}$. Thus,

$$\boldsymbol{f} = \boldsymbol{H}^{-1}\boldsymbol{z} \tag{6}$$

where $\boldsymbol{H} = \boldsymbol{I} + \lambda\boldsymbol{D}^T\boldsymbol{D}$.

Based on DCT [25, 36], (Eq 6) is reformulated as,

$$\boldsymbol{f} = \mathrm{IDCT}(\boldsymbol{\Gamma}\mathrm{DCT}(\boldsymbol{z})) \tag{7}$$

where DCT and IDCT represent the discrete cosine transform and the inverse discrete cosine transform, respectively; $\boldsymbol{\Gamma}$ is a diagonal matrix with each nonzero element being the function of the smoothing parameter $\lambda$ and eigenvalues of $\boldsymbol{D}$.

The computational cost for solving (Eq 7) is about $(mn\log(mn))/3$ [25], indicating that its performance is approximately linear with respect to the number of grids in the study domain. From the following discussion, we can see that DCT-based robust TPS should be performed with an iterative manner. Therefore, the ratio of the computational cost of the analytical TPS to that of the DCT-based robust TPS is $k^3/l\log(mn)$, where $l$ is the totally iterative times of DCT-based TPS for surface modeling. In practice, to assure the interpolation accuracy of the analytical TPS, we often set $k = 12$, and for the DCT-based robust TPS, three iterations are enough to make it convergence, i.e. $l = 3$. Based on above assumption, the ratio can be transformed to $576/\log(mn)$. Therefore, how much time the new method can save in a real case depends on the number of grids (i.e. $m\times n$) in the study domain.

Due to various reasons such as the physical limitation of sensors, low contrast of terrain textures, multiple reflectance and occlusions, outliers commonly occur in lidar point clouds [29, 30]. To reduce the influence of outliers on the construction of reference surfaces, a robust TPS is further introduced based on a pre-defined weight function with respect to fitting residuals. Supposing that $\boldsymbol{W}$ is a diagonal matrix diag($w_i$) that contains the weight $w_i\in[0,1]$ corresponding to the data $z_i$, the objective function of robust TPS is reformulated as:

$$\min_{f}(\|\boldsymbol{W}^{1/2}(\boldsymbol{z}-\boldsymbol{f})\|^2+\lambda\|\boldsymbol{Df}\|^2) \tag{8}$$

Like the deduction of (Eq 6), we can obtain the equation: $(\boldsymbol{W}+\lambda\boldsymbol{D}^T\boldsymbol{D})\boldsymbol{f}=\boldsymbol{Wz}$. Or, $(\boldsymbol{I}+\lambda\boldsymbol{D}^T\boldsymbol{D})\boldsymbol{f} = (\boldsymbol{I}-\boldsymbol{W})\boldsymbol{z}+\boldsymbol{W}_{\boldsymbol{z}}$. Thus, we obtain:

$$\boldsymbol{f}^{i+1}=\boldsymbol{H}^{-1}(\boldsymbol{W}(\boldsymbol{z}-\boldsymbol{f}^i)+\boldsymbol{f}^i) \tag{9}$$

where $i$ is the iterative times.

Based on DCT [25, 36], (Eq 9) can be expressed as

$$\boldsymbol{f}^{i+1}=\mathrm{IDCT}(\boldsymbol{\Gamma}\mathrm{DCT}(\boldsymbol{W}(\boldsymbol{z}-\boldsymbol{f}^i)+\boldsymbol{f}^i)) \tag{10}$$

It should be noted that for the grids without lidar points located in, we set $w = 0$. For the grids containing lidar points, their weights for the first iteration are set to one, and for the following iterations are defined by a bisquare weight function with respect to simulation residuals:

$$w_i=\begin{cases}\left(1-\left(\frac{r_i}{4.685}\right)^2\right)^2 & \text{for}\left|\frac{r_i}{4.685}\right|<1\\ 0 & \text{others}\end{cases} \tag{11}$$

where $r_i$ is the Studentized residual defined as, $r_i=e_i/\hat{\sigma}\sqrt{1-h}$, where $e_i$ is the simulation residual; $h$ is the leverage value, defined by $h = \mathrm{Tr}(H)/m\cdot n$; $\hat{\sigma}$ is the standard deviation of simulation residuals, computed by $\hat{\sigma}=1.483\mathrm{MAD}$, where MAD denotes the median absolute deviation of residuals [31]. The rationale behind defining (Eq 11) is that points with large simulation residuals are commonly less accurate. Thus, they should have small weights. This trick was commonly adopted in weighted least square algorithm, such as robust locally weighted regression [32]. Note that to solve Eqs 7 and 10, we use the MATLAB codes DCTN, IDCTN and SMOOTHN [25, 36] available from the MATLAB file exchange via the following links:

1. https://cn.mathworks.com/matlabcentral/fileexchange/25634-robust-spline-smoothing-for-1-d-to-n-d-data
2. https://cn.mathworks.com/matlabcentral/fileexchange/26040-dct-and-dst--+-inverse--in-arbitrary-dimension

## Procedure of the proposed method for classification

The detailed procedure of the proposed method for lidar classification is as follows (Fig 1):

(i) Select initial ground points. The minimum points in a local square window with the size of *w* are selected as the initial ground points. For almost all filters, low outliers, caused by multi-path reflections and lidar system errors, must be removed beforehand [8, 12, 16, 19]. However, this is unnecessary for the proposed method, since the effect of low outliers on the construction of reference surfaces can be reduced by the pre-defined weight function.

(ii) Cover the study domain using grids with a resolution of *h*.

(iii) Locate the selected ground points on the grids. Due to the irregular distribution of lidar points, some grids may be empty, which do not contain lidar points. They are termed empty grids. The other grids are termed known grids. When more than one point is located on the same grid, the minimum point is used.

(iv) Interpolate the grid surface by the robust TPS. Here, only the empty grids and the grids containing outliers are estimated. After the interpolation, all grids have values. The interpolated surface is used as the reference surface in step (v).

(v) Compute the elevation differences between unclassified points and the corresponding 3×3 neighboring grids in the reference surface.

(vi) Classify the lidar points as ground returns, if at least 4 out of 9 elevation differences are smaller than a pre-defined threshold *t*.

(vii) Repeat (iii)-(vi) until the maximum number of iteration is reached or no ground point is included. This process is called inner iteration.

(viii) Repeat (ii)-(vii) at the next hierarchy with $h = h/2$ and $t = t+\Delta t$. This process is called outer iteration.

# Experiments and results

Fifteen benchmark reference samples from seven sites, provided by ISPRS Commission III/WG3 [7] and downloaded from the website (https://www.itc.nl/isprs/wgIII-3/filtertest/downloadsites/), were used to assess the performances of the proposed algorithm and MHC. Note that the proposed algorithm and MHC were coded using MATLAB R2014b on a personal computer with Intel Core i7-4700 CPU @ 3.6 GHz and 8.0 GB memory. For quantitative analysis, kappa coefficient ($\kappa$), type I, type II and total errors were used as accuracy measures. Four parameters including *w*, *h*, *t* and $\Delta t$ should be pre-defined for the proposed method. Their optimal values determined by trial-and-error are shown in Table 1. Moreover, classification errors of the proposed method and MHC, and their computational costs (CCs) of executing the codes on the computer are given.

Results demonstrate that the proposed method obtains the best performance for samp42 in terms of total error and $\kappa$. This may be attributed to its low type I error, indicating that almost all ground points are correctly classified. Similar results can be also found for samp31. The proposed method has the lowest accuracy for samp53 in terms of κ, and for samp11 in terms of total error. This result can be expected, since almost all filters produced poor classification results for steep slope and discontinuities areas [7, 8, 20]. Comparatively, with respect to total error and $\kappa$, the proposed method has a better performance than MHC in almost all samples, except for samp31, samp51 and samp71. Averagely, the former produces better results than the latter. Specifically, compared with MHC, the total error of the proposed method is reduced

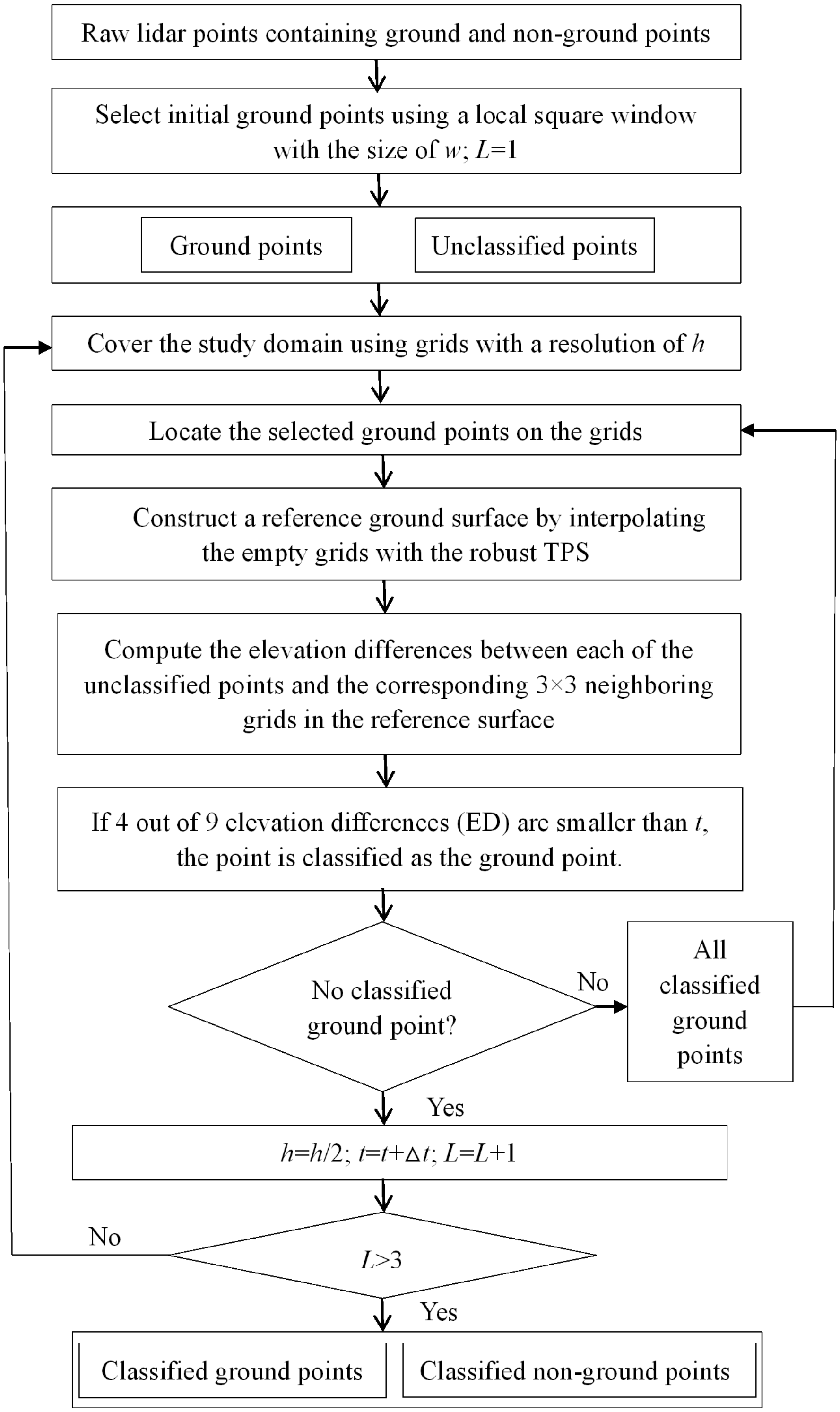


**Fig 1. Flow chart of the proposed algorithm for lidar classification.**

https://doi.org/10.1371/journal.pone.0176954.g001

**Table 1. Classification errors and computational costs (CCs) of MHC and the proposed method with the optimized parameters.** Classification errors include type I error (T.I), type II error (T.II), total error (T.E) and kappa (κ).

| sample | Optimized (m) | | | | Proposed method | | | | | MHC | | |
|---|---|---|---|---|---|---|---|---|---|---|---|---|
| | *w* | *h* | *t* | Δ*t* | T.I (%) | T.II (%) | T.E (%) | κ (%) | CC (s) | T.E (%) | κ (%) | CC (s) |
| **11** | 20 | 4 | 0.2 | 0.3 | 8.34 | 11.06 | 9.50 | 80.58 | 29 | 13.01 | 74.12 | 66 |
| **12** | 28 | 4 | 0.4 | 0.1 | 1.47 | 4.29 | 2.85 | 94.30 | 9 | 3.38 | 93.23 | 156 |
| **21** | 30 | 6 | 0.2 | 0.2 | 0.18 | 4.38 | 1.11 | 96.74 | 17 | 1.34 | 96.10 | 11 |
| **22** | 30 | 4 | 0.6 | 0.1 | 1.79 | 8.23 | 3.80 | 91.04 | 8 | 4.67 | 89.03 | 245 |
| **23** | 20 | 2 | 0.2 | 0.2 | 3.86 | 5.15 | 4.47 | 91.03 | 7 | 5.24 | 89.49 | 155 |
| **24** | 12 | 1 | 0.3 | 0.1 | 1.99 | 7.97 | 3.63 | 90.81 | 16 | 6.29 | 84.53 | 44 |
| **31** | 16 | 9 | 0.2 | 0.1 | 0.52 | 2.18 | 1.29 | 97.41 | 7 | 1.11 | 97.76 | 12 |
| **41** | 30 | 4 | 0.4 | 0.3 | 3.41 | 4.21 | 3.81 | 92.38 | 16 | 5.58 | 88.83 | 72 |
| **42** | 30 | 6 | 0.2 | 0.3 | 0.39 | 1.03 | 0.85 | 97.97 | 15 | 1.72 | 95.81 | 31 |
| **51** | 20 | 4 | 0.3 | 0.1 | 0.83 | 5.60 | 1.87 | 94.46 | 19 | 1.64 | 95.17 | 91 |
| **52** | 30 | 2 | 0.6 | 0.1 | 1.58 | 16.34 | 3.13 | 83.15 | 26 | 4.18 | 78.91 | 527 |
| **53** | 10 | 2 | 0.8 | 0.1 | 2.50 | 25.41 | 3.42 | 62.02 | 28 | 7.29 | 46.69 | 140 |
| **54** | 22 | 4 | 0.35 | 0.1 | 2.11 | 3.33 | 2.76 | 94.45 | 19 | 3.09 | 93.90 | 19 |
| **61** | 10 | 2 | 0.8 | 0.1 | 0.39 | 16.67 | 0.95 | 85.26 | 34 | 1.81 | 77.36 | 548 |
| **71** | 30 | 8 | 0.4 | 0.1 | 1.55 | 6.10 | 2.06 | 89.98 | 16 | 1.33 | 93.19 | 109 |
| **Average** | | | | | 2.06 | 8.13 | 3.03 | 89.44 | 18 | 4.11 | 86.27 | 148 |

https://doi.org/10.1371/journal.pone.0176954.t001

by 26.3% and $\kappa$ is increased by 3.7%. Computing costs of the two methods show that the proposed method is considerably more efficient that MHC for almost all samples. On average, the former is about 8.2 times as fast as the latter. The high speed of the proposed method is mainly attributed to the fast solution of TPS equations with DCT.

Here, two study sites (i.e. samp11 and samp53) with different landscapes were employed as representatives to compare the classification results of the proposed method and MHC. Samp11 is located in an urban area with low vegetation and mixed buildings on steep slopes [7]. This sample with a complex landscape often puzzled the classical filters. The type I error of MHC is much larger than that of the proposed method (Figs 2 and 3). For MHC, many terrain points on steep slopes are misclassified, such as those flagged by the rectangles (Fig 2). Comparatively, the new method preserves the shapes of discontinuous terrain features very well (Fig 3). Yet, it has a more tendency toward type II error than MHC. For example, the non-ground points marked by the ellipses are wrongly flagged as ground points (Fig 3). This may be caused by the fact that some points located on the building are selected as the initial ground points due to the small window size (i.e. 20 m) (Table 1). Considering the fact that type II errors can be more easily handled by human editing than type I errors, the inclination to type II errors may not be a shortcoming in filtering strategies.

Samp53 is located in a rural area, and mainly characterized by discontinuities and low vegetation on steep slopes [7]. Results demonstrate that the proposed method performs better than MHC for capturing ground points in terms of type I error (Figs 4 and 5). Some ground points on steep slopes are misclassified as non-ground points by MHC, such as those flagged by the rectangles (Fig 4). Comparatively, the proposed method has the ability of keeping terrain points very well at the cost of increasing type II errors, such as the points marked by the ellipses (Fig 5). According to quantitative analysis, the proposed method is much more accurate than MHC in terms of total error and $\kappa$ (Table 1).

Recently, several promising filtering methods [14, 19, 20, 33] have been developed and were assessed with the 15 groups of ISPRS benchmarks. Accuracy comparison between our method

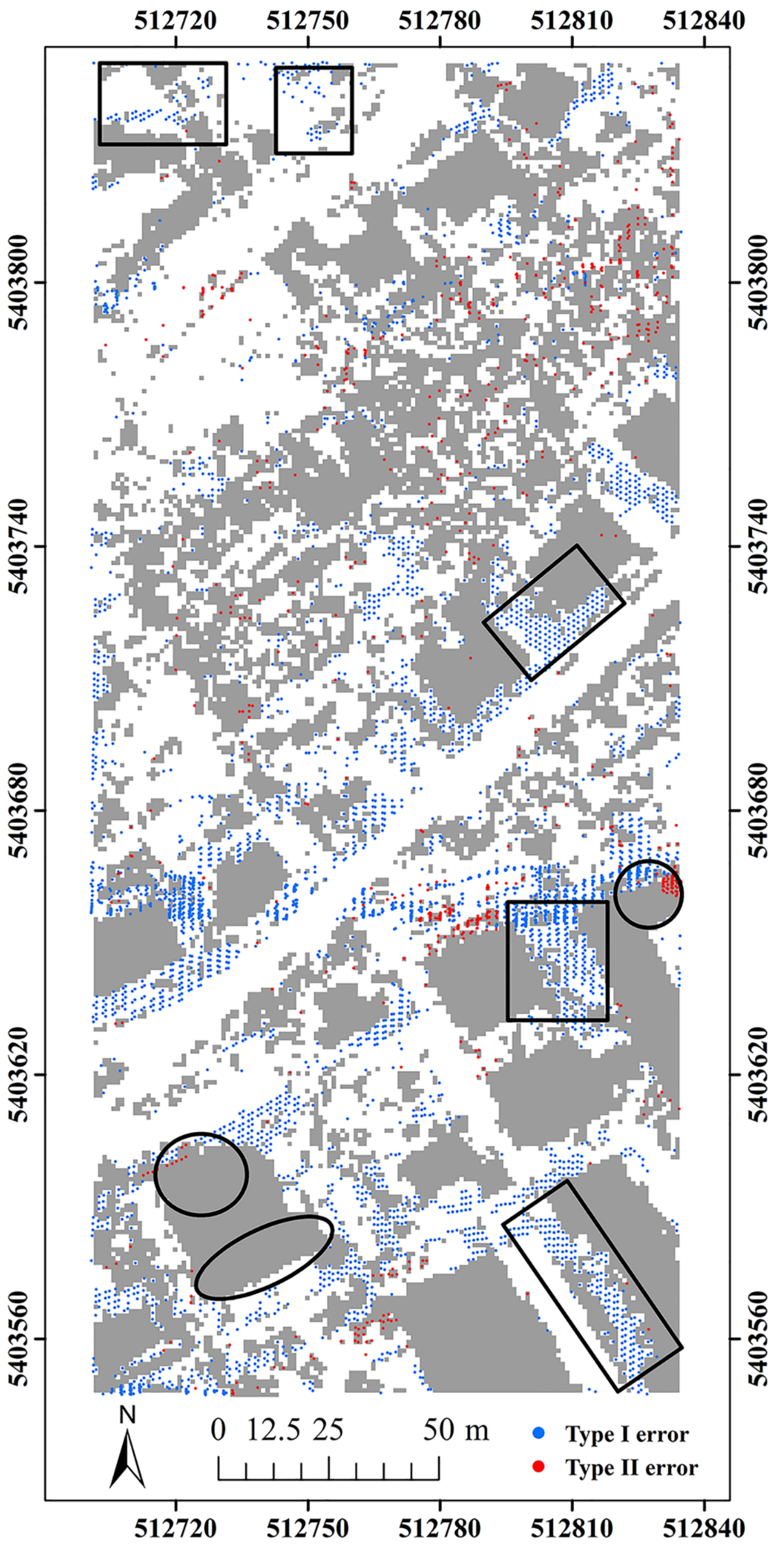


**Fig 2. Classification errors of MHC for samp11.**

https://doi.org/10.1371/journal.pone.0176954.g002

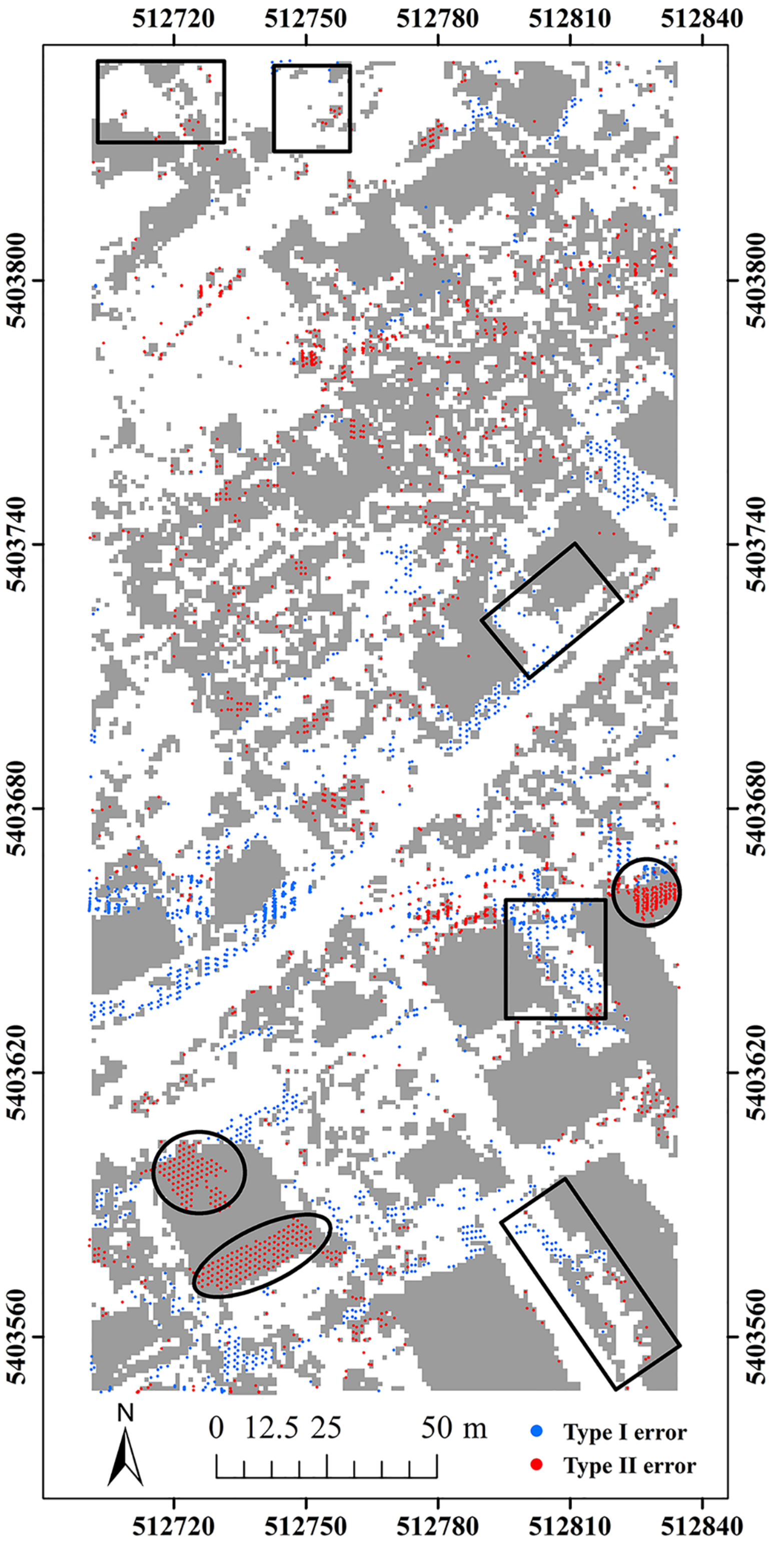


**Fig 3. Classification errors of the proposed method for samp11.**

https://doi.org/10.1371/journal.pone.0176954.g003

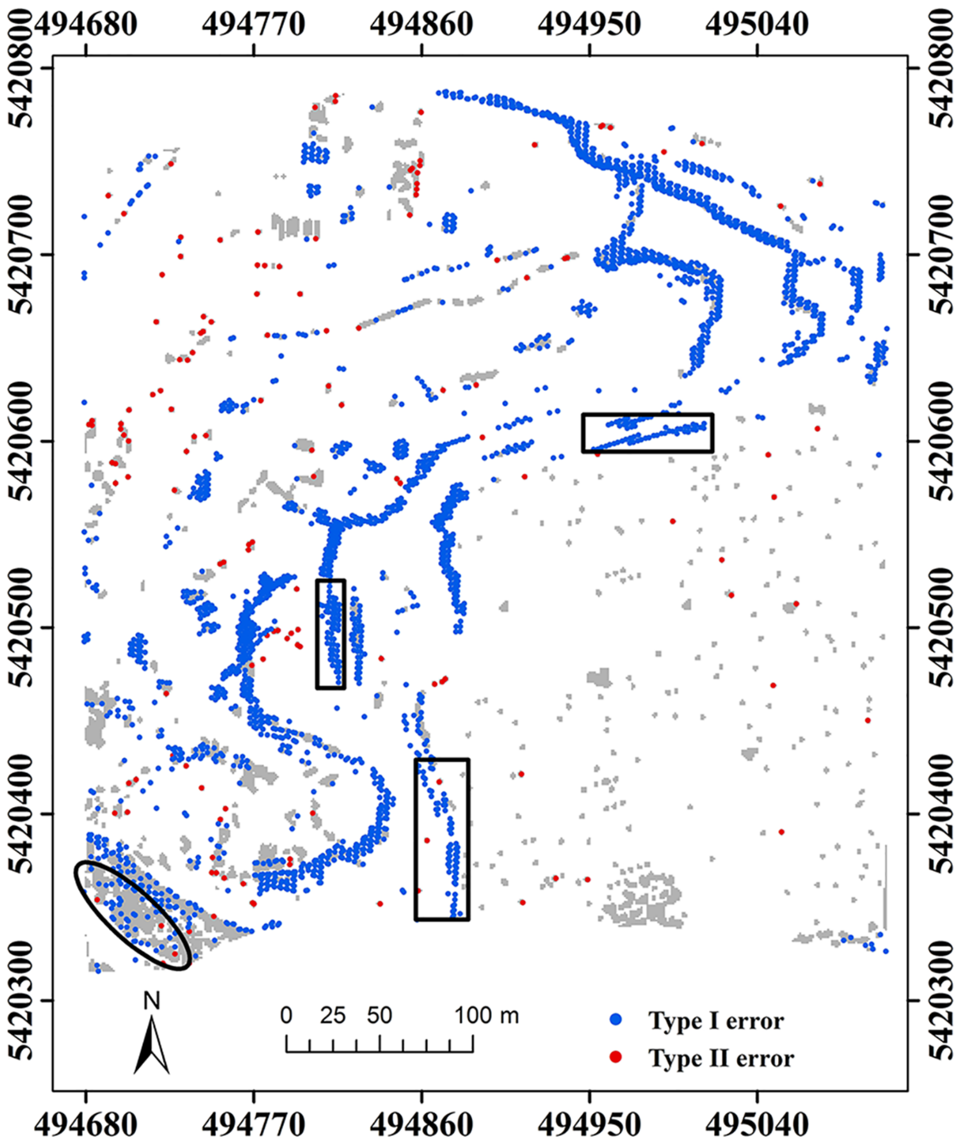


**Fig 4. Classification errors of MHC for samp53.**

https://doi.org/10.1371/journal.pone.0176954.g004

and the four newly developed methods are shown in Figs 6 and 7. It can be found that no method is consistently more accurate than the others in all samples. Our method obtains the best results for samp12, samp21 and samp24 in terms of type I error (Fig 6), and for samp41 in terms of type II error (Fig 7).

To assess the robustness of the proposed method, a series of experiments were performed with different parameters configurations, namely, $w$ ranges from 20 to 30 m with an interval of 5 m, $h$ ranges from 2 to 10 m with an interval of 2 m, $t$ ranges from 0.2 to 0.8 m with an interval of 0.1 m and $\Delta t$ ranges from 0.1 to 0.3 m with an interval of 0.1 m. Thus, there are totally 315 results for each sample. Fig 8 shows the average total error of all parameters configurations and the total error of the optimized parameters for each sample. Results show that samp41 has the largest difference between the average and optimized total errors. Specifically, the increasing ratio of the total error is about 195%. The reason may be that this site has many buildings

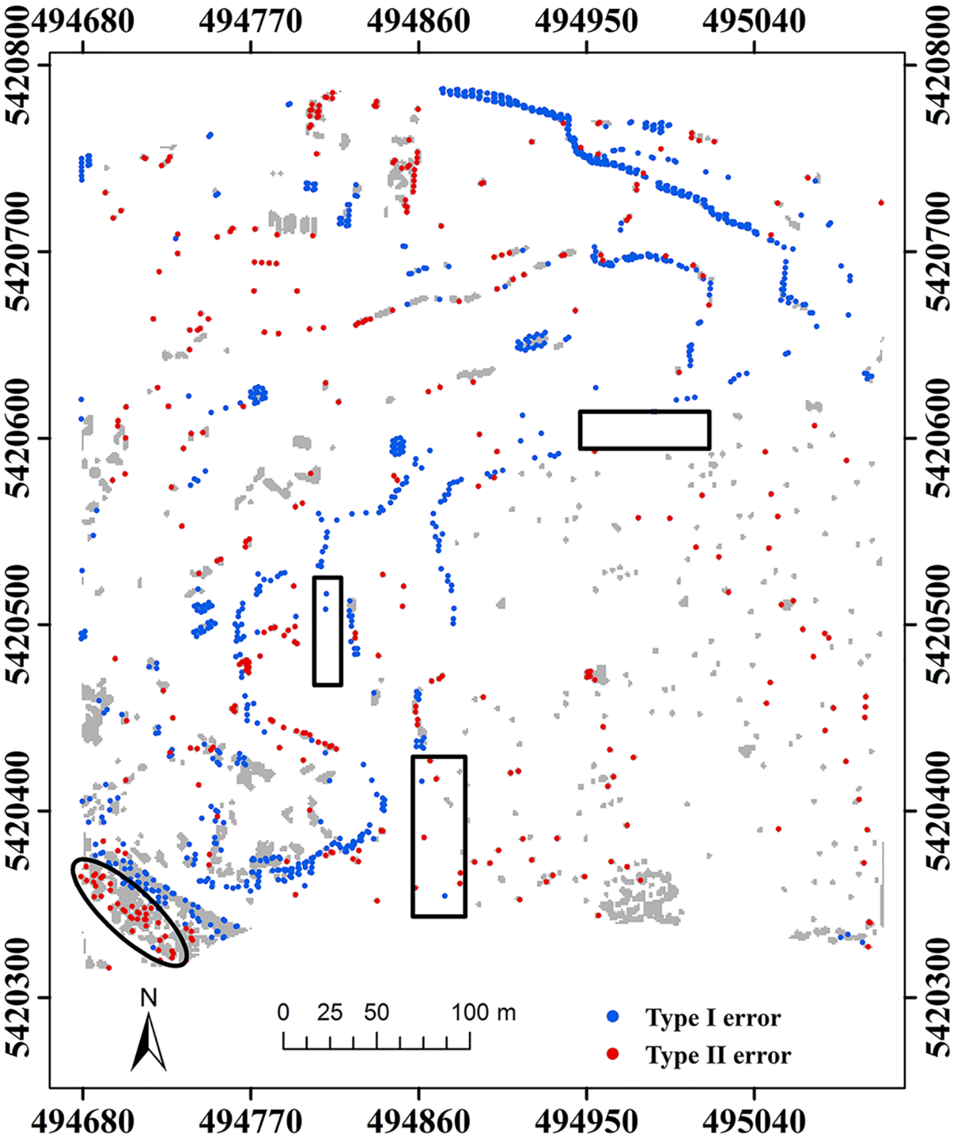


**Fig 5. Classification errors of the proposed method for samp53.**

https://doi.org/10.1371/journal.pone.0176954.g005

with the sizes larger than 20 m, and when the size of the local square window is not larger than 20 m, many non-ground points would be mixed in the initial ground points. For all samples, the average total errors range from 2.55% to 12.88% with the mean of 3.03%, while the optimized total errors from 0.85% to 9.5% with the mean of 5.88%. Therefore, the proposed method is not much influenced by the configuration of the parameters values in a reasonable range and thus obtains robust filtering results.

## Discussion

In geosciences, any sources of sample points are subject to noise. Thus, a smoothing interpolation method is more proper than an exact one for performing interpolations [34, 35]. In this paper, we used finite difference TPS for constructing reference surfaces. Here, samp21 was used to evaluate the effect of smoothness on classification, where TPS performs interpolations

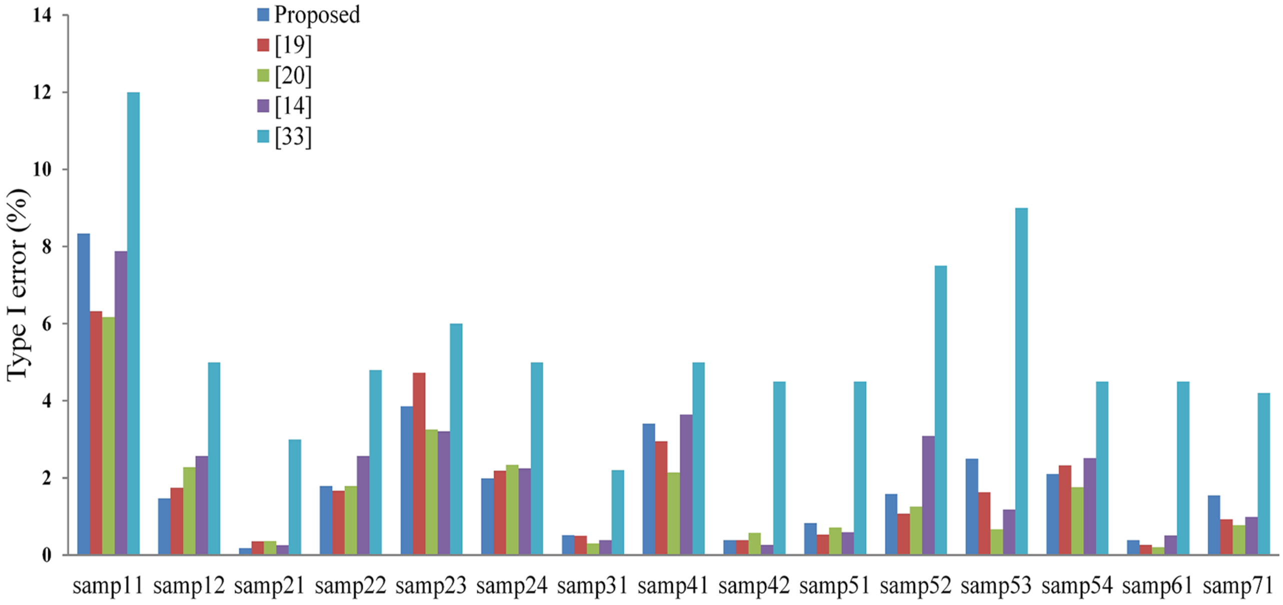


**Fig 6. Type I errors of the proposed method and the methods respectively presented by [19], [20], [14], and [33].**

https://doi.org/10.1371/journal.pone.0176954.g006

with and without the smoothing parameter. Samp21 is located in an urban area with a narrow bridge (Figs 9 and 10). It can be found that without the smoothing parameter, some points located on the bridge are misclassified as ground, as flagged by the rectangle (Fig 9). The reason for the large type II error can be explained by analyzing the initial ground points. We found that when the size of the local square window is 30 m (Table 1), one point located on the bridge is incorrectly selected as the ground seed. Thus, in the following iterations, the

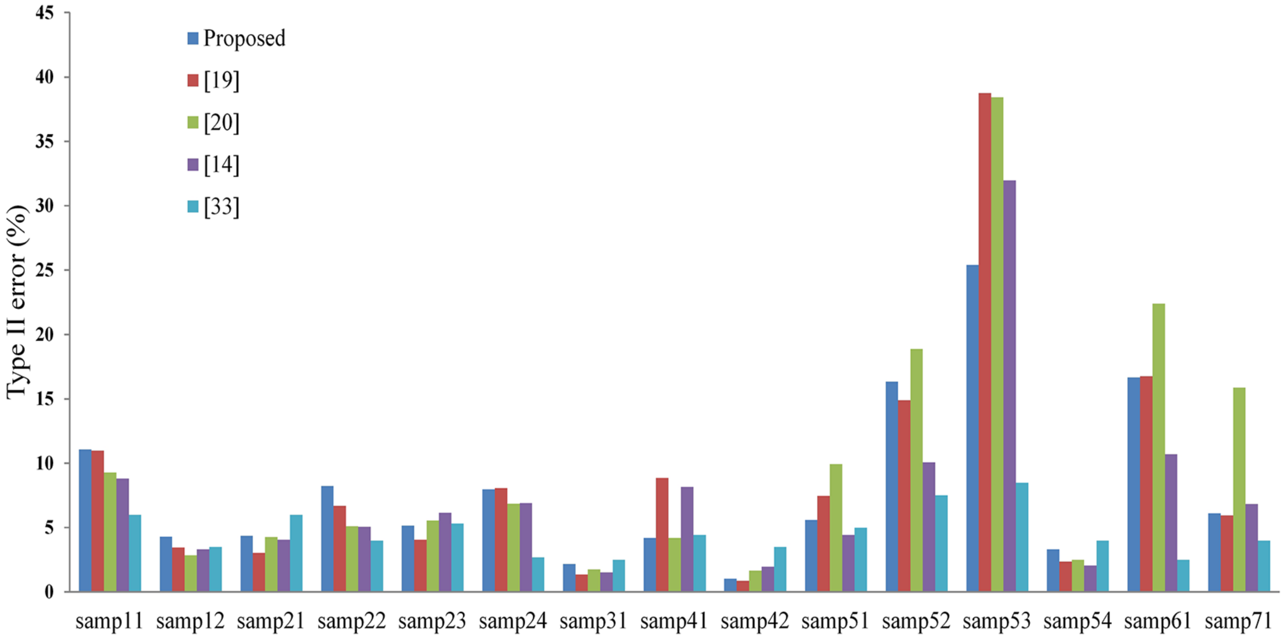


**Fig 7. Type II errors of the proposed method and the methods respectively presented by [19], [20], [14], and [33].**

https://doi.org/10.1371/journal.pone.0176954.g007

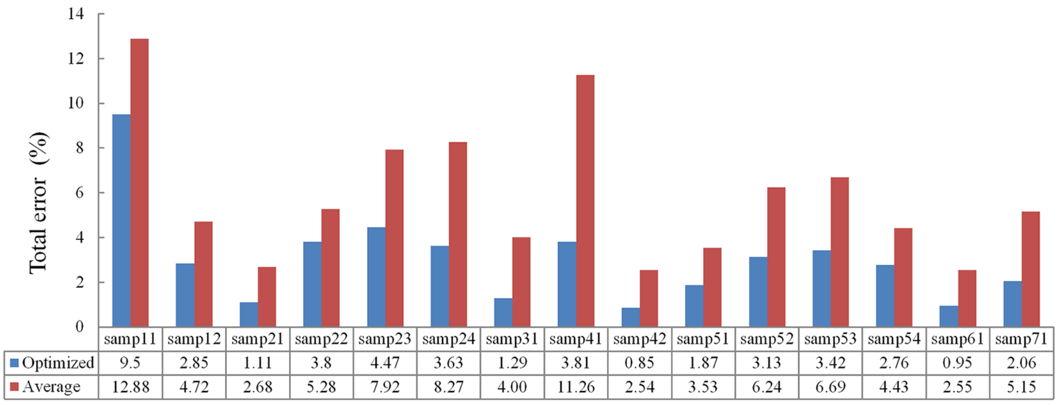


| | samp11 | samp12 | samp21 | samp22 | samp23 | samp24 | samp31 | samp41 | samp42 | samp51 | samp52 | samp53 | samp54 | samp61 | samp71 |
|---|---|---|---|---|---|---|---|---|---|---|---|---|---|---|---|
| Optimized | 9.5 | 2.85 | 1.11 | 3.8 | 4.47 | 3.63 | 1.29 | 3.81 | 0.85 | 1.87 | 3.13 | 3.42 | 2.76 | 0.95 | 2.06 |
| Average | 12.88 | 4.72 | 2.68 | 5.28 | 7.92 | 8.27 | 4.00 | 11.26 | 2.54 | 3.53 | 6.24 | 6.69 | 4.43 | 2.55 | 5.15 |

**Fig 8. Average total error of all parameters configurations and the total error of the optimized parameters for each sample.**

https://doi.org/10.1371/journal.pone.0176954.g008

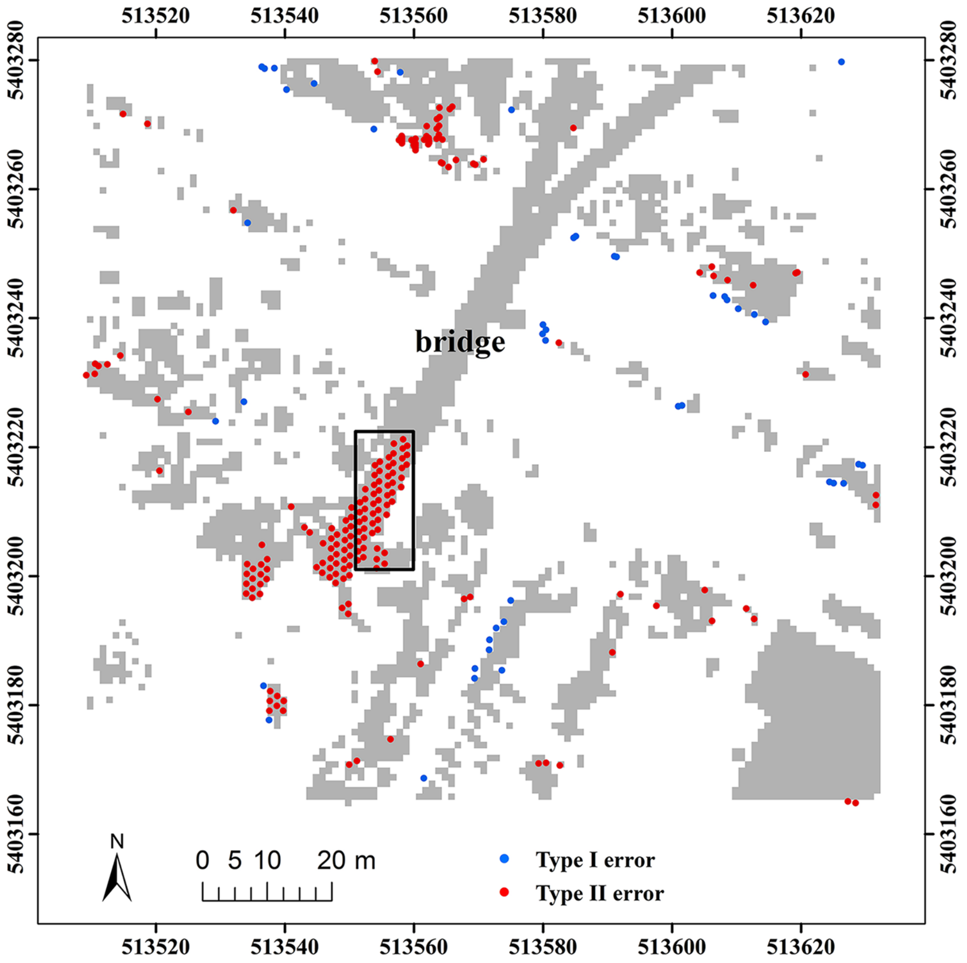


**Fig 9. Classification errors of the proposed method without the smoothing parameter for samp21.**

https://doi.org/10.1371/journal.pone.0176954.g009

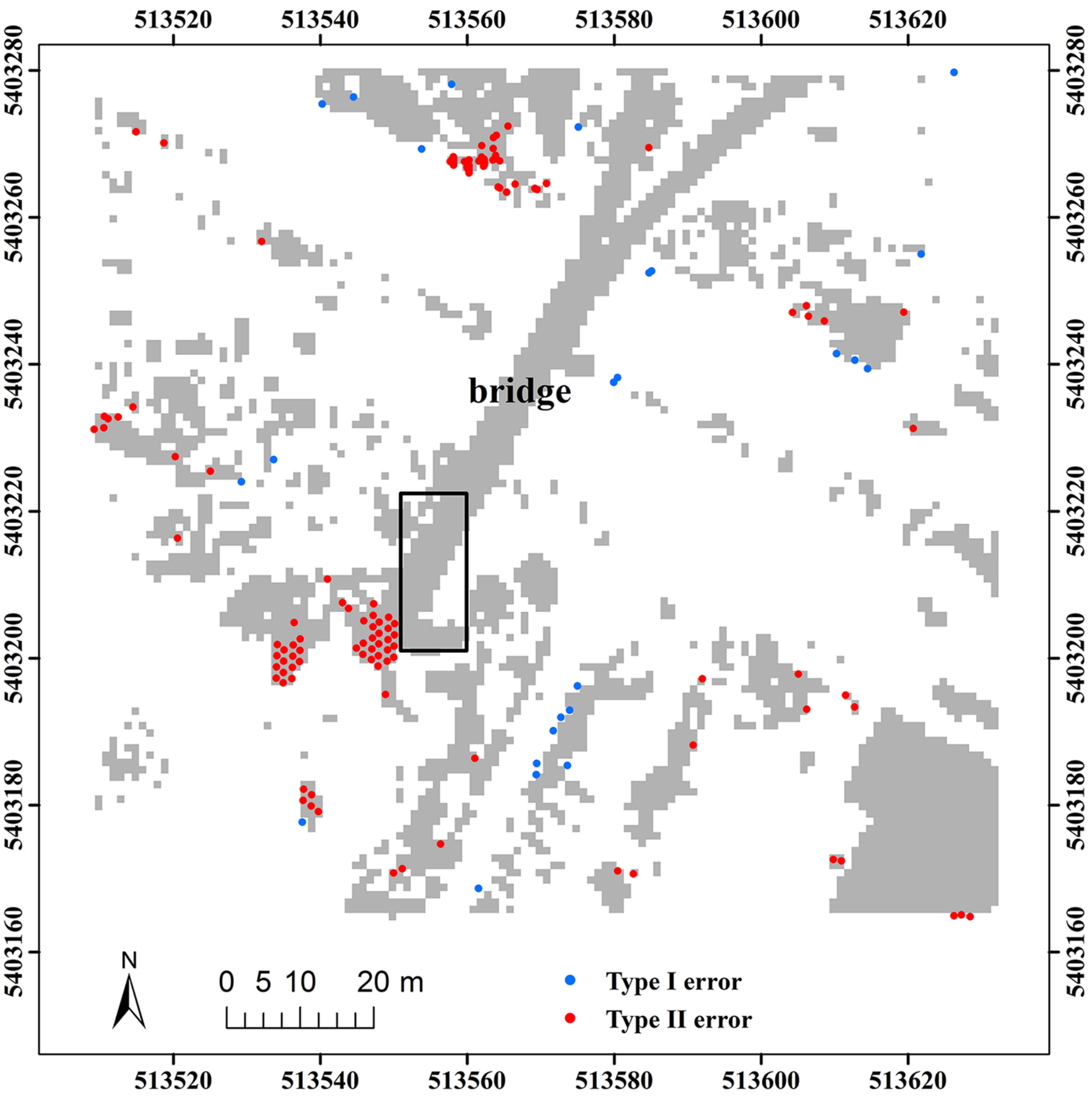


**Fig 10. Classification errors of the proposed method with the smoothing parameter for samp21.**

https://doi.org/10.1371/journal.pone.0176954.g010

misclassified point influences the accuracy of reference surfaces, which allow more non-ground points to be marked as ground. With the smoothing parameter, the negative effect of non-ground points can be resisted for surface construction in the following iterations (Fig 10).

To further assess the advantages of the robustness scheme in TPS interpolation, we employed samp22 to analyze filter results, with and without the weight function. Samp22 is located in an urban area, mainly characterized by a bridge and a gangway. Results demonstrate that only using the smoothness effect cannot completely avoid misclassification of non-ground points, such as those marked by the rectangles (Fig 11). However, when the weight function is adopted, the marked non-ground points located on the bridge and gangway are correctly labeled (Fig 12). This indirectly proves the good ability of the weight function for reducing the effect of non-ground points.

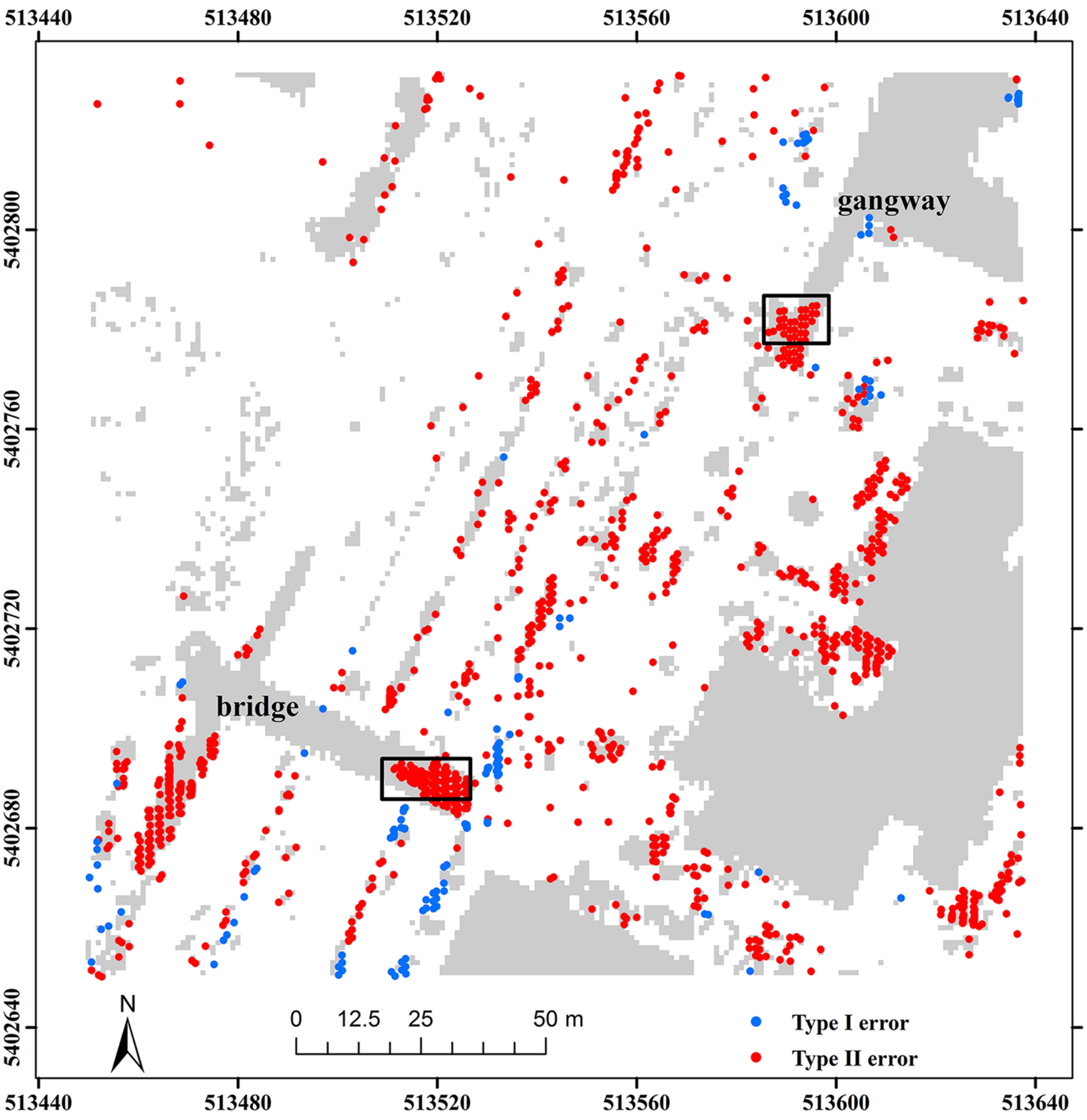


**Fig 11. Classification errors of the proposed method without the weight function for samp22.**

https://doi.org/10.1371/journal.pone.0176954.g011

Smoothing effect can result in a peak-cutting and valley-filling problem for surface construction, where subtle terrain features may be lost [19]. In our test, this shortcoming was easily overcome by replacing the simulated values of all known grids with their original ones, except for outliers. The weight function (i.e. Equation (17)) can classify points into inliers and outliers. Figs 13 and 14 show the effect of point replacement on classification errors for samp52. Samp52 is located in a rural area with low vegetation located on steep slopes and discontinuities. Results indicate that without point replacement, some ground points located on discontinuities, such as those flagged by the rectangles, are wrongly labeled as non-ground points (Fig 13). However, the above misclassification is completely avoided by the point replacement scheme (Fig 14).

From aforementioned discussion, we can see that the superior performance of the proposed algorithm to MHC can be attributed to the following facts: (i) the new algorithm uses a

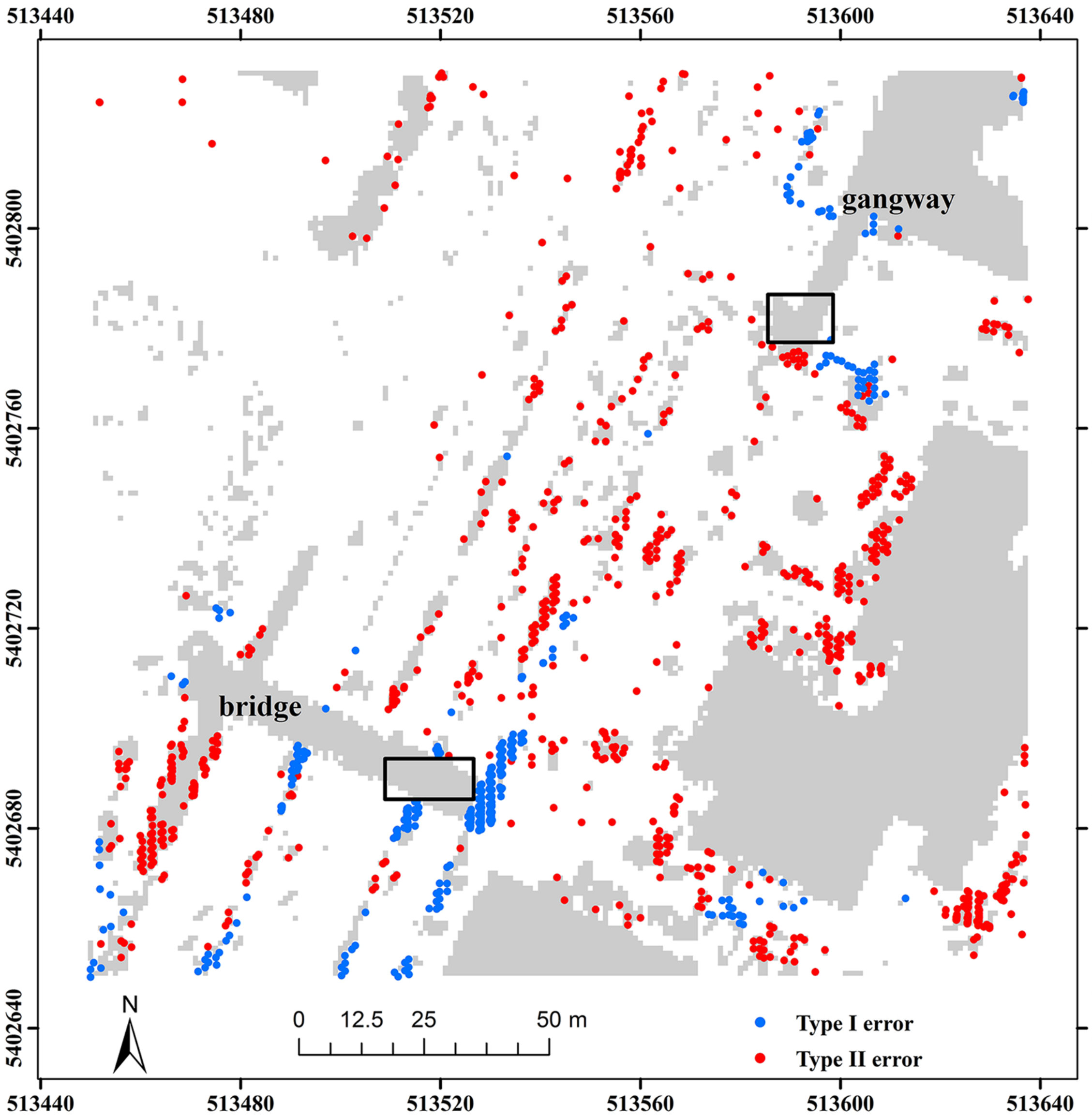


**Fig 12. Classification errors of the proposed method with the weight function for samp22.**

https://doi.org/10.1371/journal.pone.0176954.g012

smoothing parameter to remove noise inherent in sample points; (ii) it employs a weight function to reduce the influence of outliers and misclassified non-ground points on reference surface construction and (iii) it avoids the peak-cutting and valley-filling problems by replacing the fitted values with the original ones.

## Conclusions

To improve the computational efficiency of present TPS-based interpolation filters, a fast and robust filter based on finite difference TPS computation was developed in this paper. The high speed and robustness of the proposed method were respectively achieved by DCT to solve the linear system of TPS equations and by a pre-defined weight function to reduce the effect of outliers on the construction of reference surfaces. Fifteen groups of ISPRS benchmarks were employed to comparatively analyze the performances of the proposed method and MHC.

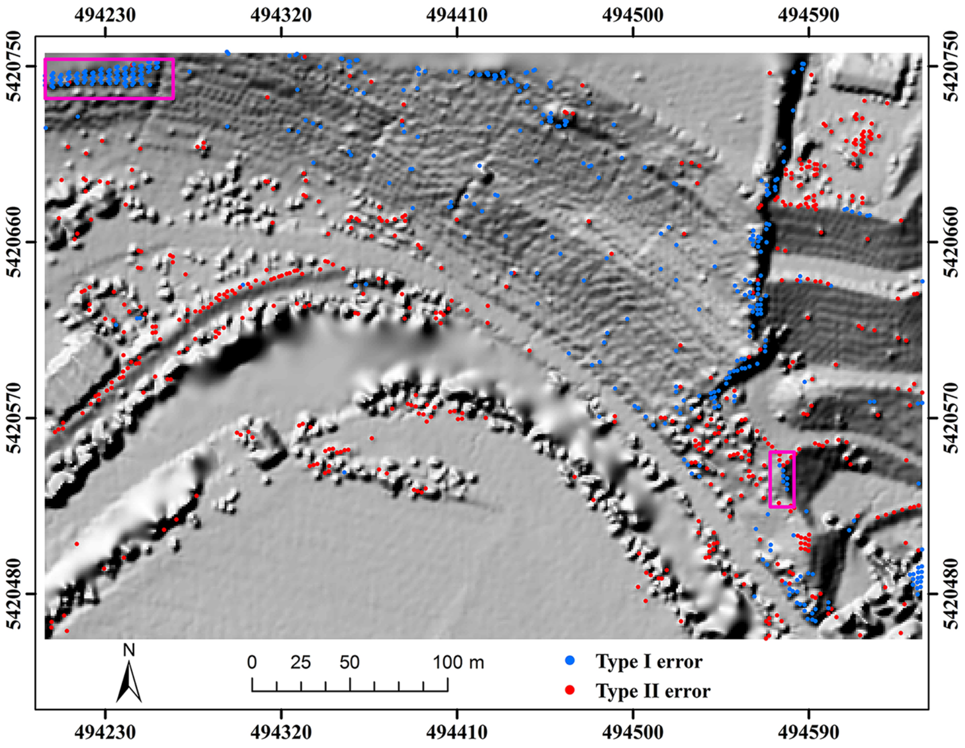


**Fig 13. Classification errors of the proposed method without point replacement for samp52.**

https://doi.org/10.1371/journal.pone.0176954.g013

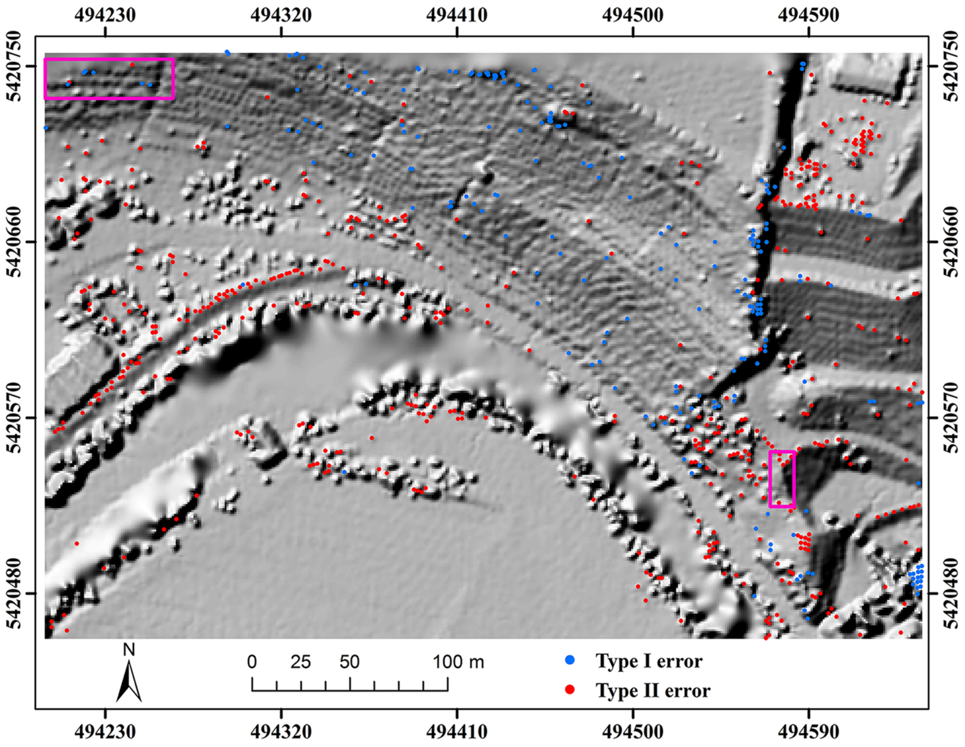


**Fig 14. Classification errors of the proposed method with point replacement for samp52.**

https://doi.org/10.1371/journal.pone.0176954.g014

Results indicated that the former was averagely much more accurate than the latter in terms of total error and kappa coefficient. Specifically, compared with MHC, the total error of the proposed method is reduced by 26%, and the kappa coefficient is increased by 3.7%. Moreover, the proposed method is about 8.2 times faster than MHC. Compared with the recently developed methods, the proposed method also obtains a good performance.

## Supporting information

**S1 Data. The fifteen benchmark reference samples provided by ISPRS Commission III/WG3.**
(RAR)

**S1 File. MATLAB codes of the proposed method.**
(ZIP)

## Author Contributions

**Conceptualization:** CC.

**Data curation:** CC YL.

**Formal analysis:** CC NZ GL.

**Funding acquisition:** CC.

**Investigation:** CC.

**Methodology:** CC YL NZ JG GL.

**Project administration:** CC.

**Resources:** CC.

**Software:** CC.

**Supervision:** CC.

**Validation:** CC.

**Visualization:** CC.

**Writing – original draft:** CC.

**Writing – review & editing:** YL JG.

## References

1. Petzold B, Reiss P, Stössel W. Laser scanning—surveying and mapping agencies are using a new technique for the derivation of digital terrain models. ISPRS Journal of Photogrammetry and Remote Sensing. 1999; 54(2):95–104.
2. Clark ML, Clark DB, Roberts DA. Small-footprint lidar estimation of sub-canopy elevation and tree height in a tropical rain forest landscape. Remote Sensing of Environment. 2004; 91(1):68–89.
3. Yang H, Chen W, Qian T, Shen D, Wang J. The Extraction of Vegetation Points from LiDAR Using 3D Fractal Dimension Analyses. Remote Sensing. 2015; 7(8):10815–31.
4. Vierling LA, Vierling KT, Adam P, Hudak AT. Using satellite and airborne LiDAR to model woodpecker habitat occupancy at the landscape scale. PloS one. 2013; 8(12):e80988. https://doi.org/10.1371/journal.pone.0080988 PMID: 24324655

5. Ji R, Gao Y, Liu W, Xie X, Tian Q, Li X. When location meets social multimedia: A comprehensive survey on location-aware social multimedia. ACM Transactions on Intelligent System and Technology. 2014; 5(2):1–20.
6. Ji R, Gao Y, Hong R, Liu Q, Tao D, Li X. Spectral-spatial constraint hyperspectral image classification. IEEE Transactions on Geoscience and Remote Sensing. 2014; 52(3):1811–24.
7. Sithole G, Vosselman G. Experimental comparison of filter algorithms for bare-Earth extraction from airborne laser scanning point clouds. ISPRS Journal of Photogrammetry and Remote Sensing. 2004; 59 (1):85–101.
8. Meng X, Currit N, Zhao K. Ground filtering algorithms for airborne LiDAR data: A review of critical issues. Remote Sensing. 2010; 2(3):833–60.
9. Chen Q, Wang H, Zhang H, Sun M, Liu X. A point cloud filtering approach to generating DTMs for steep mountainous areas and adjacent residential Areas. Remote Sensing. 2016; 8:71–92.
10. Vosselman G. Slope based filtering of laser altimetry data. International Archives of Photogrammetry and Remote Sensing. 2000; 33(Part B3/2):935–42.
11. Kilian J, Haala N, Englich M. Capture and evaluation of airborne laser scanner data. International Archives of Photogrammetry and Remote Sensing. 1996; 31(Part B3):383–8.
12. Zhang K, Chen SC, Whitman D, Shyu ML, Yan J, Zhang C. A progressive morphological filter for removing nonground measurements from airborne LIDAR data. IEEE Transactions on Geoscience and Remote Sensing. 2003; 41(4):872–82.
13. Chen Q, Gong P, Baldocchi D, Xie G. Filtering airborne laser scanning data with morphological methods. Photogrammetric Engineering & Remote Sensing. 2007; 73(2):175–85.
14. Pingel TJ, Clarke KC, McBride WA. An improved simple morphological filter for the terrain classification of airborne LIDAR data. ISPRS Journal of Photogrammetry and Remote Sensing. 2013; 77:21–30.
15. Hui Z, Hu Y, Yevenyo Y, Yu X. An improved morphological algorithm for filtering airborne LiDAR point cloud based on multi-level kriging interpolation. Remote Sensing. 2016; 8:1–35.
16. Mongus D, Žalik B. Parameter-free ground filtering of LiDAR data for automatic DTM generation. ISPRS Journal of Photogrammetry and Remote Sensing. 2012; 67:1–12.
17. Evans JS, Hudak AT. A multiscale curvature algorithm for classifying discrete return lidar in forested environments. IEEE Transactions on Geoscience and Remote Sensing. 2007; 45(4):1029–38.
18. Chen CF, Li YY, Li W, Dai HL. A multiresolution hierarchical classification algorithm for filtering airborne LiDAR data. ISPRS Journal of Photogrammetry and Remote Sensing. 2013; 82:1–9.
19. Hu H, Ding Y, Zhu Q, Wu B, Lin H, Du Z, et al. An adaptive surface filter for airborne laser scanning point clouds by means of regularization and bending energy. ISPRS Journal of Photogrammetry and Remote Sensing. 2014; 92:98–111.
20. Mongus D, Lukač N, Žalik B. Ground and building extraction from LiDAR data based on differential morphological profiles and locally fitted surfaces. ISPRS Journal of Photogrammetry and Remote Sensing. 2014; 93:145–56.
21. Hancock PA, Hutchinson MF. Spatial interpolation of large climate data sets using bivariate thin plate smoothing splines. Environmental Modelling & Software. 2006; 21(12):1684–94.
22. Zhang Y, Guan T, Duan L, Wei B, Gao J, Mao T. Inertial sensors supported visual descriptors encoding and geometric verification for mobile visual location recognition applications. Signal Processing. 2015; 112:17–26.
23. Guan T, Wang Y, Duan L, Ji R. On-device mobile landmark recognition using binarized descriptor with multifeature fusion. ACM Transactions on Intelligent Systems and Technology (TIST). 2015; 7(1):12.
24. Eilers PHC. A perfect smoother. Analytical Chemistry. 2003; 75(14):3631–6. PMID: 14570219
25. Garcia D. Robust smoothing of gridded data in one and higher dimensions with missing values. Computational Statistics & Data Analysis. 2010; 54(4):1167–78.
26. Wahba G. Spline Models for Observational Data. Philadelphia: SIAM; 1990.
27. An S, Liu W, Venkatesh S. Fast cross-validation algorithms for least squares support vector machine and kernel ridge regression. Pattern Recognition. 2007; 40(8):2154–62.
28. Chen CF, Yue TX, Dai HL, Tian MY. The smoothness of HASM. International Journal of Geographical Information Science. 2013; 27(8):1651–67.
29. Nurunnabi A, West G, Belton D. Outlier detection and robust normal-curvature estimation in mobile laser scanning 3D point cloud data. Pattern Recognition. 2015; 48(4):1400–15.
30. Sotoodeh S. Outlier detection in laser scanner point clouds. International Archives of Photogrammetry, Remote Sensing and Spatial Information Sciences. 2006; 36(5):297–302.

**31.** Rousseeuw PJ, Hubert M. Robust statistics for outlier detection. Wiley Interdisciplinary Reviews: Data Mining and Knowledge Discovery. 2011; 1(1):73–9.

**32.** Cleveland WS. Robust locally weighted regression and smoothing scatterplots. Journal of the American statistical association. 1979; 74(368):829–36.

**33.** Hu X, Ye L, Pang S, Shan J. Semi-global filtering of airborne LiDAR data for fast extraction of digital terrain models. Remote Sensing. 2015; 7(8):10996–1015.

**34.** Chen CF, Li YY, Cao XW, Dai HL. Smooth surface modeling of DEMs based on a regularized least squares method of thin plate spline. Math Geosci 2014; 46(8):909–29.

**35.** Billings SD, Newsam GN, Beatson RK. Smooth fitting of geophysical data using continuous global surfaces. Geophysics. 2002; 67(6):1823–34.

**36.** Garcia D. A fast all-in-one method for automated post-processing of PIV data. Experiments in Fluids. 2011; 50(5):1247–59. https://doi.org/10.1007/s00348-010-0985-y
